# Repeats and EST analysis for new organisms

**DOI:** 10.1186/1471-2164-9-23

**Published:** 2008-01-18

**Authors:** Ketil Malde, Inge Jonassen

**Affiliations:** 1 Computational Biology Unit, Bergen Centre for Computational Sciences, University of Bergen, Norway; 2 Institute of Marine Research, Bergen, Norway; 3 Department of Informatics, University of Bergen, Norway

## Abstract

**Background:**

Repeat masking is an important step in the EST analysis pipeline. For new species, genomic knowledge is scarce and good repeat libraries are typically unavailable. In these cases it is common practice to mask against known repeats from other species (i.e., model organisms). There are few studies that investigate the effectiveness of this approach, or attempt to evaluate the different methods for identifying and masking repeats.

**Results:**

Using zebrafish and medaka as example organisms, we show that accurate repeat masking is an important factor for obtaining a high quality clustering. Furthermore, we show that masking with standard repeat libraries based on curated genomic information from other species has little or no positive effect on the quality of the resulting EST clustering. Library based repeat masking which often constitutes a computational bottleneck in the EST analysis pipeline can therefore be reduced to species specific repeat libraries, or perhaps eliminated entirely. In contrast, substantially improved results can be achived by applying a repeat library derived from a partial reference clustering (e.g., from mapping sequences against a partially sequenced genome).

**Conclusion:**

Of the methods explored, we find that the best EST clustering is achieved after masking with repeat libraries that are species specific. In the absence of such libraries, library-less masking gives results superior to the current practice of using cross-species, genome-based libraries.

## Backgound

There is a multitude of species scientifically or commercially interesting enough to be subject to genomic research. However, as the sequencing of an entire genome is still a large undertaking, complete genome assemblies are only available for relatively few species.

In contrast, ESTs are inexpensive to produce, and even sequencing on a modest scale is likely to yield important knowledge, including information about gene transcripts [1], polymorphisms [2,3], alternative splicing [4-6], and single-nucleotide polymorphisms [2,7]. As a consequence, EST sequences constitute one of the most voluminous parts of available sequence data, and remain an important resource for analyzing transcriptomes.

To facilitate further analysis, ESTs are clustered and assembled into contigs representing gene transcripts. The goal of the clustering is to group the ESTs by originating gene. When the genome is available, genes can be identified by mapping ESTs to the genome sequence directly [8-10]. Here we will focus on the case when the genome sequence is unavailable, and clustering is performed based on sequence similarity between the ESTs.

For the similarity-based clustering to be effective, ESTs must be masked to eliminate sequence parts that would cause incorrect clustering [11]. Targets for masking include genomic repeats (sequence fragments identical to or strongly resembling fragments of other genes, e.g., due to paralogs, transposons, conserved domains, or UTR signals), vector sequence, low complexity sequence (including poly-A tails), and sequencing artifacts (e.g., from polymerase slippage), and there exists a number of methods that address the various types of repeats. Although "repeat" is often used to mean a transposon or other genomic repeat, in the context of clustering we use it to denote any similarity between unrelated sequences that would potentially lead to incorrect clustering if not masked.

For low complexity repeats, several algorithms and tools exist, including *mdust *(Tatusov and Lipman, unpublished; recently described in [12]), *DustMasker *[13] and *SeqClean *(G. Pertea, unpublished). RepeatMasker [14] is probably the most widely used tool for masking against libraries of known repeats, and it is distributed and typically used with the RepBase library [15]. RepBase contains curated repeats and is biased towards well-studied organisms. When the genome is available, species specific repeat libraries can also be constructed automatically from the genome sequence [12,16,17].

It is also possible to detect repeats directly from the EST sequences without using a pre-defined repeat library. RBR [18] is a tool that masks sequences by identifying regions of the sequence that contain words that occur more frequently in the data set than words from the surrounding regions [19]. Masked regions are thus not limited to genomic repeats, but can also be low complexity sequence or sequencing artifacts. Since the masking depends only on the ESTs themselves, it is particularly useful for identifying and masking repeats in ESTs in the absence of genomic information like repeat libraries or genome sequence. In the following we investigate the effect of different approaches to repeat masking. We develop a method for constructing repeat libraries specifically aimed at EST masking, and analyze the results obtained by using such libraries in the analysis of new EST data sets, both from the same species and from other species. The resulting clusterings are compared to those obtained using curated, genome-based repeat libraries and library-less masking methods. The results contributes to an improved basis for choosing an optimal strategy for EST sequencing and analysis.

There are several ongoing efforts to sequence different species of fish with high commercial and scientific value. Current analysis typically uses repeat libraries derived from well known species, and their performance on distantly releated species have not been accurately measured. We therfore use the available sequence information from two fish species, zebrafish (*Danio rerio*) and medaka (*Oryzias latipes*), that have both the complete genome sequence and a sizable EST collection publicly available.

## Results

For each of the two species, we randomly picked datasets containing 50 000 ESTs. Each EST was mapped to the corresponding genome and only ESTs that could be mapped unambiguously were retained (see the Methods section for details). Based on the mapping, reference EST clusters were constructed by assigning to the same cluster ESTs that mapped to overlapping genome regions.

Having constructed the reference clusters, we analysed alternative approaches to masking repeats in ESTs by clustering masked data sets with TGICL [20] and comparing to the reference. The results were quantified using two different measures; Jaccard Index and the Variation of Information (VI). The two measures reflect different features of the similarity between a clustering and a reference and we therefore included results from both (see Methods).

The alternative approaches for repeat masking were:

1. no masking (using only TGICL's built-in mdust)

2. masking using RepeatMasker and RepBase

3. library-less masking using RBR

4. masking using repeats identified from a reference clustering of one 50 K set of ESTs from the same organism or from the other organism

**Figure 1:**
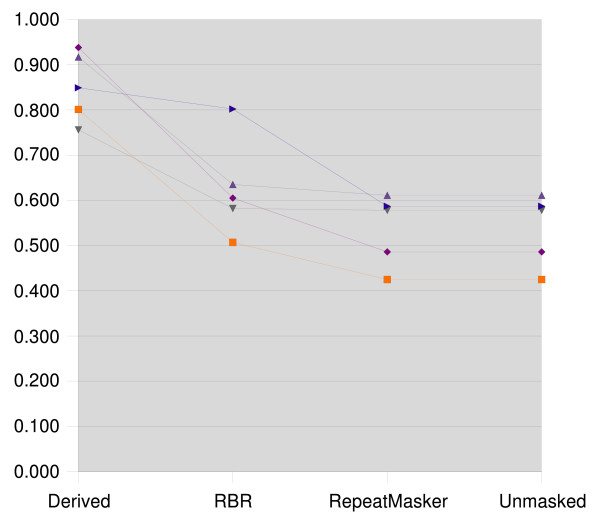
**Jaccard index for zebrafish**. Jaccard index for zebrafish, higher scores represent more accurate clusterings. In addition to the unmasked data set, each data set is masked with the repeats derived from the data set's genome-based reference clustering, with RBR, and with RepeatMasker. For clarity, the set of results for each data set are connected by lines.

**Figure 2:**
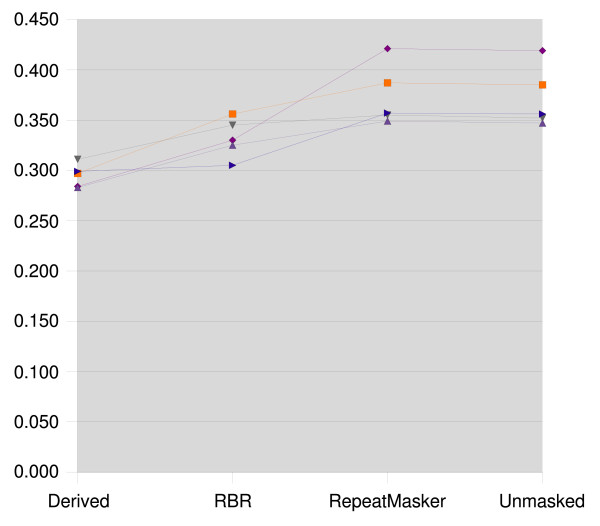
**variation of information for zebrafish**. Variation of Information for zebrafish, lower scores represent more accurate clusterings. Each line corresponds to a data set, which is masked with the repeats derived from its reference clustering, with RBR, RepeatMasker, and left unmasked.

**Figure 3:**
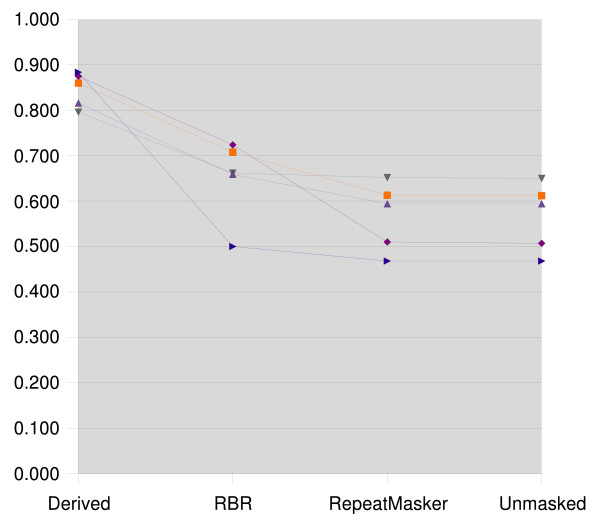
**Jaccard index for medaka**. Jaccard index for medaka, higher is better. Each line corresponds to a data set, which is masked with the repeats derived from its reference clustering, with RBR, RepeatMasker, and left unmasked.

**Figure 4:**
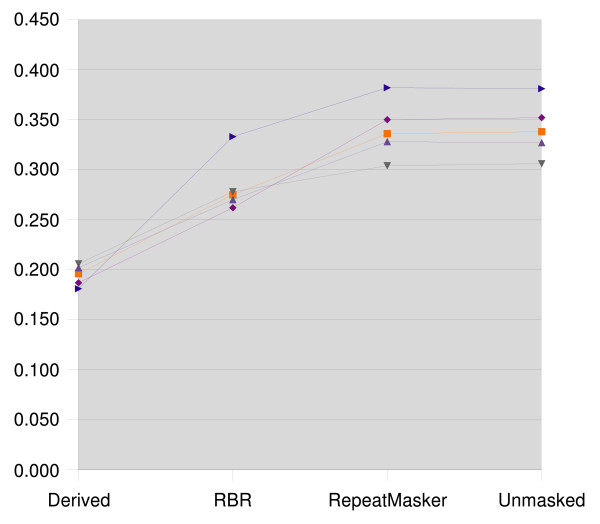
**Variation of information for medaka**. Variation of Information for medaka, lower is better. Each line corresponds to a data set, which is masked with the repeats derived from its reference clustering, with RBR, RepeatMasker, and left unmasked.

The resulting clusterings were compared to the reference clustering using the Jaccard index and the Variation of Information. The results for zebrafish are presented in Figures 1 and 2, the medaka results are presented in Figures 3 and 4.

We see that the clusterings that result from using RepeatMasker are virtually identical to clustering the unmasked sequences. RBR results in clusterings scoring between the reference derived masking and the unmasked sequences.

### Application of reference derived libraries

**Figure 5:**
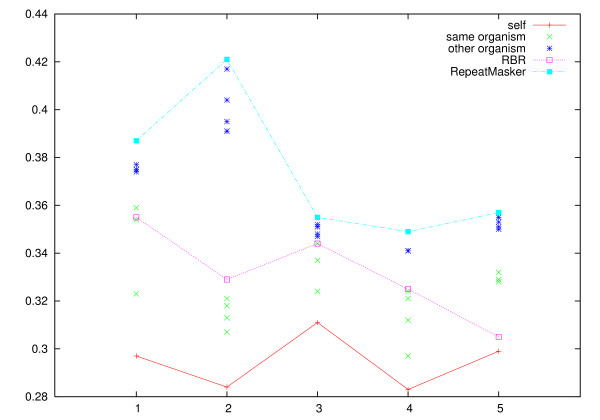
**Variation of information for zebrafish masked against other zebrafish data sets**. Variation of Information for clustering the zebrafish data sets with repeat libraries derived from the different reference clusterings. Each of the data sets (labeled 1–5) is masked with the repeat libraries derived from the reference clustering of the other zebrafish data sets, and with the reference clusterings of the repeat libraries derived from the medaka data sets. For comparison, lines show the effect of the repeat library derived from the data set's reference clustering, the RBR based masking, and masking with RepeatMasker/RepBase.

**Figure 6:**
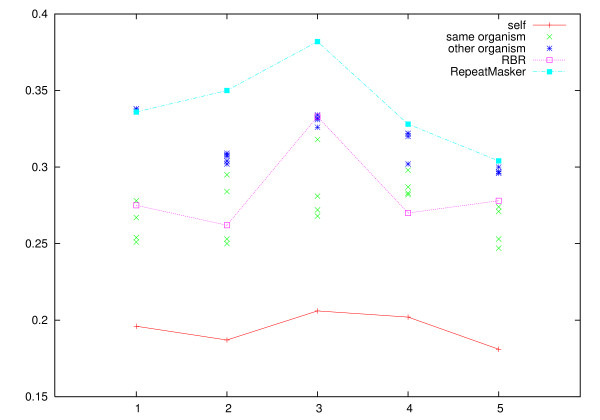
**Variation of information for medaka**. Variation of Information for clustering medaka data sets 1–5 with the derived repeat libraries. As for Figure, we show the score for masking derived from the same data set, other data sets from medaka, and zebrafish data sets, and masking with RBR and RepeatMasker/RepBase.

We wanted to investigate how well suited repeat libraries derived from one organism are for the masking ESTs from another organism and to compare this to the results obtained using a reference derived library from the same organism. Note that each reference derived library is based on one randomly picked subset of ESTs for one of the organisms. Each repeat library is then used to mask each of the other four EST subsets from that organism, and to mask the five EST subsets from the other organism. The results are summarized in Table 1 and the Variation of Information scores are shown in more detail in Figures 5 and 6.

**Table 1:** Masking results against derived libraries. Masking each data set against the libraries derived from the other data sets from the same species, and on the data sets from the other sepcies. The values are given as average Variation of Information or Jaccard index ± standard deviation.

Data sets	Libraries	VI	Jaccard
zebrafish	zebrafish	0.33 ± 0.02	0.82 ± 0.11
zebrafish	medaka	0.36 ± 0.02	0.55 ± 0.07
medaka	medaka	0.27 ± 0.02	0.68 ± 0.06
medaka	zebrafish	0.32 ± 0.02	0.59 ± 0.05

The figures show that masking sequences with a repeat library derived from a reference clustering is reasonably effective for masking other data sets from the same species. We find, however, that the reference derived libraries are consistently less effective on ESTs from another species.

## Discussion and Conclusion

EST sequences are an important resource of genomic information, and emerging high throughput sequencing technologies [21,22] have the ability to produce even larger amounts of short sequences. Sequence data generated with these technologies are likely to pose many of the same challenges as EST data, and in particular, shorter sequences are more vulnerable to repeats since a repeat potentially will cover a larger fraction of the sequence. Efficient and effective methods to address repeats are therefore a necessity.

Current repeat libraries are usually constructed for masking genomes rather than ESTs, and we do not expect these libraries to be optimal for masking ESTs. There is little reason to think that genomic repeats like transposons should be more conserved than the rest of the genome, on the contrary transposons are used in the study of closely related species or intra-species strains [23,24] as they evolve more quickly. For the organisms we have examined, we were unable to observe any merit from library-based masking using the standard, cross-species repeat libraries. This supports previous observations [18], and suggests that to be effective, repeat libraries must be based on the same or a closely related species as the sequences being masked. The execution of RepeatMasker is one of the most time-consuming parts of the pipeline, and although faster alternatives exist, the present results suggest that in the absence of a species specific library of genomic repeats, this step may be eliminated entirely with no detrimental effect on clustering quality. On the other hand, it is encouraging to see that we can obtain good results by using a repeat library derived from a genome-based reference clustering of a partial data set. This implies that the existence of a partial genome assembly not only would enable the ESTs from genes in the assembled regions to be correctly identified and analyzed, but also enable the derivation of a repeat library that can substantially improve analysis of the remaining ESTs.

Our focus has been on novel organisms where little genomic information is known. In some cases, genomes from related organisms are available, and it could then be possible to derive a reference clustering from mapping ESTs to that genome. The availability of efficient tools for automatic construction of repeat libraries from genomes also opens up the opportunity of using one or several such libraries for masking [12,16,17].

In all cases we examined, masking with RBR resulted in clusterings that are better than clustering without any masking or masked with RepeatMasker. The exact benefit varies from a modest improvement to achieving a clustering almost identical to using the library derived from the reference.

TGICL clusters sequences only when they match near the ends, with the maximal length of an acceptable non-matching "overhang" set to 30 by default. We suspect that many ESTs are not adequately trimmed for quality, and that resulting low-quality tails may produce overhangs longer than the default limit. A systematic evaluation of this parameter goes beyond the scope of this paper, but experiments increasing the overhang length to 60 yielded improved results in some cases.

We have provided both VI and the Jaccard index as a measure of cluster similarity. By adjusting RBR's parameters, it is possible to achieve better Jaccard indices at the expense of worse Variation of Information scores. In particular, less aggressive masking using Ns instead of lower case characters tends to make TGICL break up large clusters, and while it also results in more singletons, the net effect on the Jaccard index is positive. We ascribe this result to artifacts of TGICL's treatment of Ns – the TGICL manual cautions to use lower case for all masking except vector sequence – and to the Jaccard index's emphasis on large clusters. Although breaking up large, incorrect clusters will improve the efficiency of subsequent assembly, breaking up small, correct clusters will fragment the predicted genes, which is likely to be detrimental to the quality of further analysis.

In conclusion, we find that the best clustering results are achieved when ESTs are masked with repeat libraries that are species specific. In the absence of such libraries, library-less masking using RBR gives results superior to cross-species, genome-based libraries, and approaching same-species transcript-based libraries.

## Methods

### Data sets

The genome sequences for medaka and zebrafish were downloaded from Ensembl [25] version 41, and ESTs were downloaded from UniGene [26], build 94 for zebrafish and build 18 for medaka. The available EST data consisted of 1 040 346 sequences from zebrafish, and 279 369 from medaka.

To get comparably sized data sets, we chose 50 000 ESTs as a realistic size for a sequencing project for a new organism, and selected five sets of this size randomly from each organism.

### Constructing the reference clusterings

The reference clusterings were produced by matching the EST data sets with BLAT [27] against the respective genomes. As sequencing errors may result in low complexity sequence and other non-random artifacts that can lead to false matches, the genome based clustering was performed with high sensitivity, and all matches against the genome with at least 90% identity covering 75% of the length of the EST sequence were considered. In the case of multiple matches, the best match was selected. ESTs were then clustered together if their matches against the genome overlapped with at least 20 contiguous nucleotides. Some ESTs remained unmatched after this procedure. This can be caused by an incomplete or erroneously assembled genome, mitochondrial genes, contamination, or simply low quality ESTs. The unmatched sequences were discarded from further analysis. The resulting zebrafish data sets consisted of 37 894 to 38 195 sequences, while for medaka the sizes ranged from 41 919 to 42 071 sequences.

### Deriving a repeat library from a clustering

Given a (reference) clustering, it is possible to construct a corresponding repeat library consisting of the sequence fragments that occur in the sequences of multiple clusters. Although a clustering produced using such a library for masking is unlikely to be identical to the reference clustering, these sequence fragments will be the *de facto *repeats in the data set, and in this sense it represents an optimal repeat library. As our aim is not to assign any biological meaning to the derived repeats, only to eliminate them as a problem for the clustering process, repeats should be identified to the extent they would contribute to false matches in the clustering process.

TGICL uses words of length 18 to identify candidate sequences for clustering, and consequently we extracted all words of this length that occurred in two or more clusters. In the masking phase, all exact occurrences of these words in the set of sequences to be clustered were masked. The source code for the software used is available for download from [28].

To investigate the effectiveness of this masking, as well as establishing a baseline for comparing other masking methods, the masked sequences were reclustered with TGICL, and the resulting clusterings were compared to the reference. The results are summarized in Table 2.

**Table 2:** Optimality of the derived repeat libraries. Deriving a repeat library from the reference clustering of each data set, we can measure the effectiveness of the library by comparing the reclustering of the masked sequences to the reference clustering. The values are given as the average score for each species ± the standard deviation.

	VI	Jaccard
zebrafish	0.290 ± 0.01	0.85 ± 0.08
medaka	0.194 ± 0.01	0.85 ± 0.04

### Measuring similarity between clusterings

Pair-based indices, like the Jaccard and Rand indices [29], are commonly used to compare similarity between different clusterings. The Jaccard index is defined as $\frac{a}{a+b+c}$ where *a *is the number of pairs that are clustered together in both clusterings, and *b *and *c *are the numbers of pairs clustered together in one of the clusterings, but not in the other. For identical clusterings, *b *and *c *are zero and the Jaccard index reaches its optimum of 1.

Note that although *b *and *c *can be considered Type I and Type II errors [30], a misclustered sequence will inflate these numbers in proportion to the number of pairs the sequence generates – i.e., with the sizes of the clusters containing the sequence. This means that for the pair-based indices, the composition of large clusters will be disproportionally more important than the composition of smaller ones.

Previously, we have supplemented Jaccard scores with an entropy-based measure called *Variation of Information *[18,31] as a supplement to the Jaccard index. Similar to Jaccard, larger clusters affect the Variation of Information more than small ones, but the effect is less emphatic than for pair-based indices. The Variation of Information reaches its optimum of 0 when the clusterings are equal.

Although the measures have different emphasis, comparison of two clusterings that are very similar should result in a good score using either measure. In the following, we therefore provide both the Jaccard index and the Variation of Information.

### Masking and clustering

For clustering, we used TGICL [20], using the -X parameter to omit the assembly stage, which is irrelevant for this comparison. TGICL incorporates *mdust *for low complexity filtering, and *megablast *[32] for aligning and scoring sequences. By default, TGICL requires exact matches of length 18 to identify candidate sequence pairs.

For masking sequences, we used RepeatMasker (using the -xsmall option), and RBR [18] version 0.8 with default options.

## Authors' contributions

KM and IJ conceived the idea and designed the analyses together. KM performed the analyses and drafted the paper. IJ contributed to the finalisation of the manuscript. Both authors have read and approved the final version.
